# One is the loneliest number: Involuntary celibacy (incel), mental health, and loneliness

**DOI:** 10.1007/s12144-023-04275-z

**Published:** 2023-02-02

**Authors:** Brandon Sparks, Alexandra M. Zidenberg, Mark E. Olver

**Affiliations:** 1 Kingston University, 55-59 Penrhyn Rd., Kingston Upon Thames, KT1 2EE UK; 2 King’s University College at Western University, London, Canada; 3 University of Saskatchewan, Saskatoon, Canada

**Keywords:** Incel, Involuntary celibacy, Dating, Loneliness, Misogyny, Violence against women

## Abstract

Incels—a ragtag collection of young males who have rallied around their shared experience of romantic rejection—have slowly emerged as an online group of interest to researchers, no doubt as a result of several high-profile attacks. Much of this work has centered around incels’ dating experiences, sexual attitudes, and online forums. However, it is possible that their moniker, short for involuntary celibate, has resulted in an overemphasis on their sexual exclusion and frustration. Recent work has identified social isolation as a key aspect of inceldom, which may help explain why incels have responded negatively to romantic rejection. The present study thus sought to examine the role of social support and loneliness in experiences of rejection in a sample of incel (*n* = 67) and non-incel (*n* = 103) men. Results indicated that incels experience more feelings of loneliness and less social supports than non-incel men. Both of these variables were associated with multiple mental and relational health issues that incels also scored more highly on. Further, incels reported using more solitary and problematic coping mechanisms. These results suggest that incels may be missing a key buffer in sheltering them from the adverse effects of romantic rejection. It also extends previous findings highlighting the importance of attachment styles in differentiating incels from non-incels, which may perpetuate feelings of isolation. Implications for how this may relate to incel discourse and clinical interventions are discussed.

**Supplementary Information:**

The online version contains supplementary material available at 10.1007/s12144-023-04275-z.

## Introduction

Since 2014, there have been several violent incidents committed by involuntary celibates which have claimed the lives of nearly fifty people (Hoffman et al., 2020). One of the most noteworthy attacks took place on April 23, 2018, when Alek Minassian drove a rental van through downtown Toronto, killing ten people and injuring sixteen others (Mandel, 2018). Minassian paid homage to fellow incel (a portmanteau of involuntary celibate), Elliot Rodger, who killed six people and himself during a series of violent attacks in Isla Vista, California in 2014 (Duke, 2014). On the one hand, Rodger has been heralded as a martyr and the “supreme gentleman” of the incel community for the attack and the dissemination of his manifesto, in which he framed his violence as “retribution” for women having denied him the opportunity to have sex (Byerly, 2020; Jaki et al., 2019; Mandel, 2018). On the other hand, incel endorsement of Rodger and other incels who have engaged in public acts of violence is relatively low (Moskalenko et al., 2022; Speckhard et al., 2021).

This lack of sexual experience is, as their name suggests, one of the hallmarks of the involuntary celibacy community. However, the meaning of the term incel has changed drastically since it entered the public vernacular in the late 1990’s when a woman named Alana began a website for people who struggled to form romantic or sexual relations (Alana, 1997; Kassam, 2018). Today, incel has become synonymous with angry young men who seek refuge in anti-feminist online forums where they can espouse their misogynistic beliefs steeped in sexual entitlement and biological determinism (Ging, 2019; Hoffman et al., 2020; Maxwell et al., 2020). The twisted ideology behind the attacks combined with the accessible nature of most incel forums has generated considerable media and, later, research coverage. While the news coverage of incel-perpetrated violence is largely sensational, Byerly’s (2020) analysis of 70 news articles—which spanned 29 outlets in six countries—suggests that the content is an accurate portrayal of incel behaviours, ideologies, and the community as a whole. Indeed, Byerly’s claim that online communities promote of violence and misogyny is supported across multiple content analyses of several incel forums (Baele et al., 2021; Jaki et al., 2019; Maxwell et al., 2020; O’Malley et al., 2020).

That increased attention is being directed toward the incel community is promising. So, too, is Byerly’s (2020) finding that many of the journalists adopted a feminist approach in their coverage of incel violence, calling out the vitriolic attitudes that drove the behaviour. The sexual entitlement expressed by incels is extremely concerning given its natural extension to female subjugation and the bevy of research linking such expectations to violent reactions when they are not met (Blake et al., 2018; DeLecce et al., 2017). Emerging evidence even suggests that these effects can be heightened in men who are high in social dominance orientation, a measure of support for group-based hierarchies which is congruent with incel ideologies of re-establishing male social dominance (Ging, 2019; Jones, 2020; Kelly et al., 2015; Woerner et al., 2018). While misogyny and sexual rejection have understandably formed the bulk of the narratives surrounding incels, often lost in these discussions is that incels are not—despite their name—exclusively rejected sexually; rather, sexual rejection is just one of many forms of social exclusion that they experience.

### Loneliness and social isolation

In 2018, the moderators of incels.me (now incels.co) conducted a poll in which roughly 300 incels participated. When asked if they had friends, only one-third of the 294 respondents indicated that they did (Jeltsen, 2018). A lack of stable friendships has also been noted in the manifestos of Rodger and Chris Harper-Mercer, a lesser-known incel whose mass shooting was one of the deadliest in Oregon’s history (Flaccus, 2017; Rodger, 2014). In the opening of his manifesto, Harper-Mercer (2015) summed up his life as “one lonely enterprise… with no friends” (p. 1). Feelings of isolation resulting from a lack of friends (among other things) are common discussion topics among incels. A recent textual analysis of 100 discussion threads on the incels.me forum identified loneliness as one of the top 1,000 keywords, with the authors concluding that this constitutes a core aspect of inceldom (Jaki et al., 2019). This may help explain why only 18% of incels in Sparks et al. (2022) reported having pictures with friends in their dating application profile compared to 52% of non-incel men. In fact, this was the only photo category that evidenced a sizeable difference between the groups in the study.

Further analyses lend credence to the magnitude of this issue. A thematic content analysis of 834 posts in the r/Braincels subreddit identified social isolation as one of the overarching themes (Maxwell et al., 2020). Many incels lamented that they are misunderstood and unfairly labelled as sex-hungry when they also seek friendship and general social inclusion. One commenter clarified:

“incels aren’t just after sex… what they really want is affection and a genuine emotional bond. Some say that they wouldn’t care about sex as long as they could experience love. Some even say that they would be happy if they could just have platonic love instead of romantic love (some incels have very few friends or none at all).” (p. 1864).

This desire for a “genuine emotional bond” appears to stand in stark contrast to the misanthropic and indeed violent ideology that is espoused by the incel community. Yet incels are so distraught by their social isolation that they experience suicidal thoughts and question whether they would be missed if they acted on them (Maxwell et al., 2020). Jones’ (2020) own qualitative analysis of incel posts found that discussion of isolation is nested in broader conversations of depression—with self- and formal diagnoses being considerably higher in the incel community compared to the general population; see Moskalenko et al., 2022). In Jones’ (2020) work, one user noted that depression is a “natural state for ugly people [i.e., incels]” (p. 65). When incels discussed how they coped with their depressive feelings, the mechanisms (e.g., studying, reading, watching TV, lifting weights) were almost exclusively solitary, which Jones (2020) commented may exacerbate their feelings of depression and loneliness. Interestingly, several users in Maxwell et al. (2020) analysis expressed gratitude for the incel community and its role in facilitating social connections while also serving as a place to express their frustrations and support one another.

The exclusion and loneliness experienced by incels is viewed as an extension of the looks-based hierarchy on which they believe society operates (Jones, 2020; Maxwell et al., 2020). Thus, the lookism principles apply not only to their ability to attract mates, but also friends. Similarly, their social anxiety and supposed autism is frequently listed as one of the causes for incels’ romantic and social isolation (Jaki et al., 2019). Further, forty percent of the incels in the above incels.me poll specifically identified autism or other similar conditions as contributing to their isolation (ADL, 2020). While incels frequently use autism as a catch-all term for social shyness or awkwardness—and thus estimates of autism with this community should be taken rather cautiously—a recent survey indicated that 18% of incels have a formal diagnosis of autism (more than double the rate among the general population; Moskalenko et al., 2022). This was dwarfed by the 74% who were self-diagnosed with autism, suggesting either professional underdiagnosis among this population or self-overdiagnosis among incels. Regardless, what is concerning is not the cause of incels’ social isolation, but rather, its consequences (Baele et al., 2021; Jaki et al., 2019; Williams & Arntfield, 2020).

If incels are experiencing general social isolation, it may be exacerbating a number of mental and relational health issues that appear to be prominent in this community. Incels report greater rejection sensitivity and proneness to interpersonal victimhood than comparable males, suggesting that the high rates of rejection they experience may be particularly onerous on their well-being (Costello et al., 2022a; Sparks et al., 2022). Indeed, 95% of incels surveyed through incels.co reported having depression (38% had a formal diagnosis; Moskalenko et al., 2022). Both Sparks et al. (2022) and Costello et al. (2022a) also found that incels experienced greater depressive symptoms than non-incel men. Similarly, higher rates of general anxiety (Costello et al., 2022a; Moskalenko et al., 2022) and dating anxiety (Sparks et al., 2022) have been noted in this population. These have been found to relate to fears about being single which incels, not surprisingly, experience a great deal of (Sparks et al., 2022). Incels also endorse less secure attachment styles, although it is difficult to determine whether this is influenced by the magnitude of their rejection, the misogynistic content that is present on incel forums, or something else (Baele et al., 2021; Jones, 2020; Sparks et al., 2022). It is, however, consistent with depictions of women as untrustworthy and manipulative, a prominent trope in incel dialogue (Baele et al., 2021; Jaki et al., 2019; Jones, 2020).

### Social connections as buffers for adverse events

The need for social connection has been documented for centuries; Aristotle described humans as “political animals” and over 2,000 years later researchers have begun exploring the capacity for friendship between humans and artificially intelligent robots (Archer, 2021; Aristotle, 2013). In the interim, a bounty of evidence has accumulated documenting the benefits of social connections ranging from increased happiness, mental health, and cardiovascular health to the pursuit of goals (Bartolini et al., 2013; Gore, 2014; Lombardi et al., 2019; Xia & Li, 2018). On the contrary, loneliness has been associated with an increased risk of depression, pain, fatigue, inactivity, and mortality (Domènech-Abella et al., 2017; Hawkley et al., 2009; Jaremka et al., 2013; Tanskanen & Anttila, 2016). Further, experiencing loneliness can perpetuate a cycle of increased loneliness, wherein the isolated person experiences an exaggerated response to negative social events while also perceiving positive events as less pleasant (Aframn & Kashdan, 2015; Cacioppo & Hawkley, 2005).

Social supports also serve a protective purpose. Adams et al. (2011) found that receiving distressing news with a best friend present buffered the impact on participant cortisol levels and subjective measures of self-worth compared to those who received the news alone. Social support was also associated with better emotional functioning and less stressful reactions two months after receiving a terminal cancer diagnosis (Ringdal et al., 2007). Henrich and Shahar (2008) found that social support can cushion against depressive symptoms in adolescents who were exposed to rocket attacks in Israel. The amount of time spent with friends during adolescence has even been associated with protective effects in young adulthood. Specifically, adolescents who spent more time with friends demonstrated less activity in the anterior insula and dorsal anterior cingulate cortex—regions linked with the processing of pain and negative affect—after experiencing exclusion in a virtual ball-tossing game (Masten et al., 2012). Recently, Schacter et al. (2019) found that the link between peer rejection in middle school and later relational aggression was moderated by friendship quality in high school, with rejection only predicting aggression among individuals with low friendship quality during the ninth grade. Social support has also been implicated as a protective factor in the aftermath of divorce, with perceived social support mediating the relationship between loss and psychological well-being (Kołodziej-Zaleska & Przybyła-Basista, 2016). A meta-analysis of 21 studies also found that social relationships are associated with lower levels of maladjustment and higher levels of positive adjustment in recent divorcees (Kramrei et al., 2007). Potentially due to the efficacy of friendship, Perilloux and Buss (2008) found that one of the most frequently reported coping strategies among recently separated individuals was talking with friends.

### Current study

It is apparent that friendship and social support are not only integral parts of the human experience but are instrumental in buffering the effects of negative events. Given the prevalence of seeking comfort from friends when relationships go sour (and the buffering effect that is has), one must wonder whether incels’ reported lack of friends has influenced the way in which they perceive and respond to romantic rejection (Kołodziej-Zaleska & Przybyła-Basista, 2016; Kramrei et al., 2007; Perilloux & Buss, 2008). Seemingly, lacking social connections removes a potential therapeutic outlet for their relational and sexual strife, with which they must find other means of coping. According to Jones (2020), the coping mechanisms employed by incels are primarily solitary activities, which could exacerbate their feelings of isolation. What remains unclear is whether lacking social outlets for the venting of romantic/sexual frustrations is uniquely predictive of inceldom.

Thus, the aim of this study is to identify whether incels do indeed have lower levels of social support and whether that is uniquely predictive of incel identity. Similarly, social support’s role as a predictor of incel status will be tested. The study will also serve as an opportunity to replicate some of the group differences that emerged between incel and non-incel men as well as associating these measures with the recently developed Incel Traits Scale (Scaptura & Boyle, 2020), which would provide further evidence of the measure’s convergent validity. If the proposed study is successful in replicating the results of Sparks et al. (2022) or in associating these variables with the Incel Traits Scale, how perceived social support may influence the differences/associations in the fear of being single, attachment, depressive symptoms, and self-esteem can be explored. If these seemingly integral components of the incel experience are indeed a product of low levels of perceived social support, this would suggest that perceived social support may serve as both a cause of and solution to inceldom, warranting further inquiry.

### Hypotheses

As noted above, the aim of the current study is to replicate some of the findings from Sparks et al. (2022) and to identify how social isolation and a lack of social supports may be implicated in the incel experience. In accordance with this, the following hypotheses have been generated:

1. Incels will report more depressive and anxious symptoms, greater fear of being single, lower levels of social support and self-esteem, higher levels of loneliness, and endorse more problematic coping strategies relative to non-incels. Incels will also demonstrate a pattern of less secure attachment.
2. Incels will score higher on measures of social dominance orientation, externalization of blame, self-critical rumination, belief in female sexual deceptiveness, and sexual entitlement and report their perceived mate value as lower compared to non-incels.
3. The above-mentioned measures will significantly correlate with the Incel Traits Scale in the same direction as suggested in hypotheses 1–2; incels will also score higher than non-incels on this measure.
4. Perceived social support and loneliness will account for a significant portion of the differences and associations predicted in hypotheses 1 and 2.

## Method

### Sample

The study utilized an undergraduate student participant pool (SONA; restricted to students enrolled in first-year psychology courses) and a university-wide online forum (personal access to web service; PAWS) to recruit a sample that served as a comparison group to incels; men were the primary reference comparison group and the basis for testing the above hypotheses. This recruitment took place at an institution situated in the Canadian prairies. As Reddit has removed several of the exclusive incel forums, incels were recruited through study advertisements posted on related subreddits, mostly r/Virgin and to a lesser extent r/Antifeminists. Incel status was based on whether participants classified themselves as an incel or not; self-identified incels recruited via the SONA and PAWS system were included in the incel group rather than the male comparison group. Those who completed the survey through SONA were given course credit as compensation for their participation (others received no compensation). This resulted in a sample consisting of 67 incels and a comparison group of 103 non-incel males, which exceeded the minimum number of participants (34 and 96, respectively) needed to detect a moderate effect size (*d* = 0.50) with 0.80 power based on an a priori one-tailed power analysis in G*Power3 (Faul et al., 2007). However, this calculation was based on an expected allocation ratio (0.36) similar to Sparks et al. (2022), which was surpassed in the current study (0.65); as such, a sensitivity analysis revealed that effect sizes of 0.39 and above could be reported with 0.80 power. Both incels and non-incels were largely heterosexual (92.5% and 82.5%, respectively), of European ancestry (67.3% and 56.3%, respectively), in their mid-twenties (*M*_*AGE*_ = 26.83, *SD* = 11.24; *M*_*AGE*_ = 23.54, *SD* = 6.60, respectively), and were politically neutral (*M* = 4.18, *SD* = 1.45; *M* = 4.34, *SD* = 1.57). Over twice as many incels reported currently using a dating app compared to non-incel males (46% and 20%, respectively). See Table 1 for further demographic details.

**Table 1:** Participant demographics

	Full sample (*n* = 170) *M* (*SD*)/*n* (%)	Incels (*n* = 67) *M* (*SD*)/*n* (%)	Non-incels (*n* = 103) *M* (*SD*)/*n* (%)
Age	24.83 (8.84)	26.83 (11.24)	23.54 (6.60)
Sexual orientation			
Heterosexual Homosexual Bisexual Pansexual	147 (86.5%) 8 (4.7%) 12 (7.1%) 3 (1.8%)	62 (92.5%) 0 (0%) 3 (4.5%) 2 (3.0%)	85 (82.5%) 8 (7.8%) 9 (8.7%) 1 (1.0%)
Ethnicity			
African Caribbean Central American East Asian European Indigenous Middle Eastern South American South Asian Other	7 (4.1%) 1 (0.6%) 5 (2.9%) 10 (5.9%) 100 (58.8%) 11 (6.5%) 12 (7.1%) 4 (2.4%) 19 (11.2%) 10 (5.9%)	3 (4.5%) 0 (0.0%) 0 (0.0%) 3 (4.5%) 44 (65.7%) 2 (3.0%) 6 (9.0%) 1 (1.5%) 5 (7.5%) 7 (10.4%)	4 (3.9%) 1 (1.0%) 5 (4.9%) 7 (6.8%) 56 (54.4%) 9 (8.7%) 6 (5.8%) 3 (2.9%) 14 (13.6%) 3 (2.9%)
Political orientation			
Very conservative Conservative Slightly conservative Neutral Slightly liberal Liberal Very liberal	4 (2.4%) 21 (12.4%) 28 (16.5%) 41 (24.1%) 33 (19.4%) 33 (19.4%) 10 (5.9%)	1 (1.5%) 7 (10.4%) 14 (20.9%) 21 (31.3%) 9 (13.4%) 11 (16.4%) 4 (6.0%)	3 (2.9%) 14 (13.6%) 14 (13.6%) 20 (19.4%) 24 (23.3%) 22 (21.4%) 6 (5.8%)
Education level			
Less than high school degree	2 (1.2%)	2 (3.0%)	0 (0.0%)
High school or equivalent (GED)	16 (9.4%)	6 (9.0%)	10 (9.7%)
Some college but no degree Associate degree Bachelor’s degree Graduate/ professional degree	85 (50.0%) 6 (3.5%) 38 (22.4%) 23 (13.5%)	25 (37.3%) 5 (7.5%) 14 (20.9%) 15 (22.4%)	60 (58.3%) 1 (1.0%) 24 (23.3%) 8 (7.8%)

### Measures

#### State adult attachment measure

To capture the secure, anxious, and avoidant attachment styles, the State Adult Attachment Measure (SAAM; Gillath et al., 2009) was used. The items with the top three factor loadings on each domain were used, resulting in a 9-item measure. Each item is scored on a 7-point Likert-type scale ranging from 1 (*disagree strongly)* to 7 (*agree strongly*), with scores on each attachment style ranging from 3–21. Sample items include “I feel loved,” “I feel a strong need to be loved unconditionally right now,” and “I’m afraid someone will want to get too close to me,” reflecting secure, anxious, and avoidant attachment styles, respectively. Cronbach’s alphas for secure (α = 0.91), anxious (α = 0.87), and avoidant (α = 0.82) attachment were all at or above appropriate thresholds.

#### Hospital anxiety and depression scale

To measure depressive and anxious symptoms, the 14-item Hospital Anxiety and Depression Scale (HADS; Zigmond & Snaith, 1983) was used. Designed as a screening tool, the HADS has been extensively validated across several populations (Honarmand & Feinstein, 2009; Pais-Ribeiro et al., 2007) and is comprised of two 7-item scales measuring anxious and depressive symptoms. Sample items include “I feel tense or ‘wound up’” and “I feel as if I am slowed down,” reflecting anxiety and depression, respectively. Respondents report the frequency in which they experience each item using a 0–3 scale, with higher scores indicating greater frequency. Internal consistency for the depression (α = 0.77) and anxiety (α = 0.80) subscales were similar and moderate.

#### Fear of being single scale

Concern with being or experiencing singlehood was assessed using Spielmann et al.'s (2013) 6-item Fear of Being Single Scale. Using a 1 (*not at all true*) to 5 (*very true*) scale, participants responded to questions such as “I feel anxious when I think about being single forever.” Higher scores reflect greater fear of being single. Overall, the six items demonstrated good internal consistency (α = 0.89).

#### Single-item self-esteem scale

To measure participant self-esteem, Robins et al.’s (2001) Single-item Self-esteem Scale was used. Participants respond to the prompt “I have high self-esteem” using a 7-point scale ranging from 1 (*not very true of me*) to 7 (*very true of me*).

#### Externalization of blame scale

To determine the extent to which participants blame others when women reject them, the 4-item Externalization of Blame Scale (Kelly & Aunspach, 2020) was used. The scale uses a 7-point Likert-type scale ranging from 1 (*strongly disagree*) to 7 (*strongly agree*), with higher scores indicating greater externalization of blame. Sample items include “when a girl rejects me, it’s because she’s a bitch/must be frigid” and “I feel that when a girl doesn’t reciprocate my advances, it’s because she’s playing hard to get.” This scale demonstrated modest levels of internal consistency (α = 0.82).

#### Short social dominance orientation scale

Participants’ support for group dominance and hierarchies was assessed using the Short Social Dominance Orientation Scale (SSDO; Pratto et al., 2013). This 4-item measure was created as a more efficient alternative to the traditional Social Dominance Orientation (Pratto et al., 1994) measure while also addressing positive skew that was associated with some items in the original scale. Responses were recorded using a 10-point scale ranging from 1 (*extremely oppose*) to 10 (*extremely favor*). Sample items include “we should not push for group equality” and “superior groups should dominate inferior groups.” Internal consistency in this measure bordered on moderate (α = 0.69).

#### Mate value scale

Edlund and Sagarin’s (2014) 4-item mate value scale was used to determine the subjective value participants place on their own quality as a prospective mate. Participants responded to questions such as “Overall, how good of a catch are you?” using a 7-point Likert-type scale with higher scores reflecting greater desirability. This scale had a high degree of internal consistency (α = 0.92).

#### Social and emotional loneliness scale

To express the degree of emotional and social loneliness experienced by participants, the 6-item Social and Emotional Loneliness Scale (De Jong Gierveld & Van Tillburg, 2006) was used. Adapted from their longer 11-item scale, the shortened version devotes three items to both social and emotional loneliness. These include “There are plenty of people I can rely on if I have problems” and “I experience a general sense of emptiness,” respectively. Responses are recorded using a 5-point scale, ranging from 1 (*Yes!*) to 5 (*No!*), with lower scores reflecting greater loneliness. Reliability analyses indicated the scale had moderate (α = 0.71) internal consistency.

#### Sexual entitlement subscale

The degree to which one feels entitled to sex was measured using Sexual Entitlement Subscale of Widman and McNulty’s (2010) Sexual Narcissism Scale. The sexual entitlement subscale is comprised of five items reflecting one’s sexual expectations and supposed deservedness of sex. Using a 5-point scale ranging from 1 (*strongly disagree*) to 5 (*strongly agree*), participants responded to questions such as “I am entitled to sex on a regular basis,” with higher scores reflecting greater sexual entitlement. This scale demonstrated good internal consistency (α = 0.81).

#### Self-critical rumination scale

Self-critical rumination was measured using the 10-item Self-Critical Rumination Scale (Smart et al., 2016). Responses to questions like “I often berate myself for not being as productive as I should be” are recorded using a 4-point scale ranging from 1 (*almost never*) to 4 (*almost always*). The 10-item scale demonstrated strong internal consistency (α = 0.92).

#### Incel traits scale

The recently developed Incel Traits Scale (Scaptura & Boyle, 2020) was used to assess the extent participants identify with incel-related traits. Comprised of two subscales, defeated (15 items) and hateful (5 items), each item reflects an incel characteristic with its antonym flanked on the end of a 9-point scale, which was modified to a 100-point slider for the survey platform. Sample items include the following word pairings: rejected/accepted, merciful/vengeful, and calm/enraged. Overall, the scale demonstrated a high degree of internal consistency (α = 0.95).

#### Brief cope scale

The Brief Cope Scale (Carver, 1997) was used to assess 14 different coping strategies one may utilize when experiencing romantic rejection. The Brief Cope Scale is comprised of 28 items (two per strategy) and is measured on a 4-point scale (0 = *I haven’t been doing this at all*; 3 = *I’ve been doing this a lot*) based on the frequency in which they engage in each tactic. Sample items include “I’ve been taking action to try to make the situation better” (active coping) and “I’ve been making jokes about it” (humour).

#### Multidimensional scale of perceived social support

The friends and family subscales from Zimet et al.’s (1988) Multidimensional Scale of Perceived Social Support was used to measure the degree of social support participants feel that they receive from each group. As incels are single, the significant other subscale was not used in the study. This resulted in an 8-item measure split evenly between the two remaining social support groups. Answered on a 7-point scale ranging from 1 (*very strongly disagree*) to 7 (*very strongly agree*), participants responded to questions such as “my family really tries to help me” and “I have friends with whom I can share my joys and sorrows.” Reliability analyses indicated the scale had a high degree of internal consistency (α = 0.92).

#### Belief in female sexual deceptiveness scale

To determine the endorsement that women are sexually manipulative, the 14-item Belief in Female Sexual Deceptiveness Scale (Rogers et al., 2015) was used. Using a 7-point scale (0 = *never*; 6 = *almost always*), participants responded to questions such as “women enjoy toying with men’s feelings” and “women date men simply for the material benefits they can get.” This demonstrated a high level of internal consistency (α = 0.95).

#### Demographics

To properly describe the sample, a series of demographic questions were used. This included questions pertaining to the gender, age, ethnicity, sexual orientation, political orientation, incel identification, and education level. Whether they are currently using a dating application was also assessed.

### Procedure

Upon signing up for the online study, participants were presented with a consent form outlining the purpose and content of the study. If after reading the consent form they wished to participate, they were taken to the study where they filled out the demographic items followed by the remainder of the above measures in random order. After completion of the survey, a debriefing form was given that provides greater detail on the purpose of the study, articles for further reading, and how to contact the investigators. Participants took roughly twenty minutes on average to complete the study.

### Data analytic strategy

Following the data screening, coding of items, and summation of their respective constructs, the following analyses were used to test the study’s several hypotheses:

1. To identify potential group differences between incel and non-incel males, MANOVAs were conducted (see Supplemental Materials for MANCOVAs with demographics entered as covariates). This included the following dependent measures: attachment depression, anxiety, self-esteem, self-critical rumination, externalization of blame, social dominance orientation, perceived mate value, loneliness, coping strategies, sexual entitlement, perceived social support, and belief in female deceptiveness. Cohen’s *d* and partial eta squared effect sizes were reported and the former interpreted using Cohen’s (1992) guidelines where 0.20, 0.50, and 0.80 constitute the benchmarks for small, medium, and large effect sizes, respectively.
2. Correlational analyses were conducted to determine the associations between the Incel Traits Scale and all of the variables mentioned in the above analysis. These were also interpreted using Cohen’s (1992) recommendations, which suggest that correlations of 0.10, 0.30, and 0.50 reflect small, medium, and large effects, respectively.
3. To determine the role of perceived social support and loneliness in the relationship between incels and depression, anxiety, attachment, self-esteem, mate-value, externalization of blame, and self-critical rumination, a binomial logistic regression was run to determine predictors of incel group membership.

## Results

A MANOVA was used to test between-groups differences on several mental and relational health variables (see Table 2). The pattern of results largely mimicked those found in Sparks et al. (2022), again with moderate to large effects. Interestingly, significant differences between incels and non-incels emerged for all three attachment styles in this replication. Of note, this study utilized a measure of general anxiety, rather than dating-related anxiety as in Sparks et al. (2022), which was also higher among the incel participants.

**Table 2:** Mental and relational health responses

	Incels	Non-incels			
	*M*(SD)	*M*(SD)	*F*	*d*	*n*_*p*_^2^
Fear of being single	25.23 (4.93)	15.92 (6.60)	81.281***	1.55	0.371
Depression	16.52 (4.18)	12.58 (3.65)	34.703***	1.02	0.201
Anxiety	18.43 (4.52)	15.83 (4.27)	11.850***	0.59	0.079
Self-esteem	2.55 (1.76)	4.36 (1.59)	39.783***	1.09	0.224
Attachment					
Secure	7.93 (3.81)	15.77 (4.06)	131.731***	1.98	0.488
Anxious	15.80 (5.21)	13.65 (4.47)	6.797*	0.45	0.047
Avoidant	11.48 (5.06)	8.17 (4.05)	18.363***	0.74	0.117

*** *p* < 0.001; ** *p* < 0.01; * *p* < 0.05

Overall, it appears as though incels may have fewer outlets to express their romantic and sexual frustrations to than non-incel men, which may be related to the former’s greater endorsement of self-critical rumination practices (see Table 2). Analyses of coping strategies revealed some differences between groups, namely that incels utilized positive reframing and emotional support significantly less and engaged in more negative coping strategies such as behavioural disengagement and self-blame. Significant differences emerged between incel and non-incel men on measures of blame externalization and sex-related antisocial attitudes (see Tables 3, 4 and 5); however, with the exception of female sexual deceptiveness, these were of the smallest (significant) effects found in the group comparisons.

**Table 3:** Responses to rejection, date-related attitudes, and social supports

	Incels	Non-incels			
	*M*(SD)	*M*(SD)	*F*	*d*	*n*_*p*_^2^
Perceived mate value	10.58 (4.85)	19.70 (3.72)	138.935***	2.19	0.528
Externalization of blame	7.93 (5.02)	9.74 (4.35)	4.475*	0.39	0.035
Self-critical rumination	32.82 (6.45)	26.62 (7.45)	22.035***	0.87	0.151
Perceived social support	25.22 (11.69)	40.35 (10.68)	54.285***	1.37	0.304
Loneliness	24.33 (3.33)	18.59 (4.18)	62.630***	1.47	0.336

*** *p* < 0.001; ** *p* < 0.01; * *p* < 0.05

**Table 4:** Coping strategy endorsement

	Incels	Non-incels			
	*M*(SD)	*M*(SD)	*F*	*d*	*n*_*p*_^2^
Active coping	4.94 (1.96)	5.56 (1.83)	3.258	0.33	0.025
Planning	5.56 (2.09)	5.79 (1.92)	0.396	0.12	0.003
Positive reframing	4.31 (2.16)	5.38 (1.72)	9.626**	0.56	0.070
Acceptance	6.33 (1.72)	6.31 (1.51)	0.007	0.01	0.000
Humour	4.73 (2.36)	5.22 (2.02)	1.584	0.23	0.012
Religion	2.98 (1.54)	3.41 (1.94)	1.702	0.24	0.013
Emotional support	2.90 (1.26)	4.35 (1.85)	23.201***	0.88	0.154
Instrumental support	3.98 (2.03)	4.42 (1.92)	1.520	0.22	0.012
Self-distraction	6.50 (1.76)	5.81 (1.76)	4.558*	0.39	0.035
Denial	3.08 (1.47)	2.86 (1.63)	0.583	0.14	0.005
Venting	4.92 (1.77)	3.63 (1.47)	19.657***	0.81	0.134
Substance use	4.06 (2.35)	3.38 (1.88)	3.264	0.33	0.025
Behavioural disengagement	5.08 (1.99)	3.11 (1.57)	39.009***	1.13	0.235
Self-blame	6.54 (1.68)	5.23 (1.92)	15.335***	0.71	0.108

*** *p* < 0.001; ** *p* < 0.01; * *p* < 0.05

**Table 5:** Antisocial Attitudes

	Incels	Non-incels			
	*M*(SD)	*M*(SD)	*F*	*d*	*n*_*p*_^2^
Sexual entitlement	11.26 (5.11)	9.09 (3.82)	7.453**	0.50	0.056
Social dominance orientation	15.85 (7.88)	12.84 (6.56)	5.396*	0.43	0.041
Belief in female sexual deceptiveness	58.64 (15.99)	43.36 (14.49)	30.636***	1.01	0.196

*** *p* < 0.001; ** *p* < 0.01; * *p* < 0.05

As fewer participants completed all items of the Incel Traits Scale, separate univariate ANOVAs were used to compare the responses between the incel and non-incel groups. A large effect was found with incels (*M* = 1219.08, *SD* = 315.61) scoring significantly higher (*F*_(117)_ = 72.331, *p* < 0.001, *d* = 1.40, *n*_*p*_^2^ = 0.382) than non-incels (*M* = 690.06, *SD* = 403.44) on the overall measure. Incels also scored higher than non-incels on the measure’s two subscales, defeated (*M* = 879.56, *SD* = 223.18; *M* = 486.33, *SD* = 228.93, respectively) and hateful (*M* = 349.31, *SD* = 121.15; *M* = 201.93, *SD* = 122.10, respectively). These differences were both significant and of large effect (*F*_(121)_ = 79.826, *p* < 0.001, *d* = 1.73, *n*_*p*_^2^ = 0.397; *F*_(128)_ = 44.366, *p* < 0.001, *d* = 1.21, *n*_*p*_^2^ = 0.257, respectively). Higher levels of incel traits were also strongly correlated with several variables, including fear of being single, depressive and anxious symptoms, perceived social support, and loneliness (see Table 6). Contrary to expectations, loneliness and perceived social support did not account for any unique increase in predicting group membership (non-incels = 0, incels = 1) when entered into a binomial logistic regression with other mental and relational well-being measures (see Table 7). Only perceived mate value and avoidant attachment significantly predicted group membership, with the overall model classifying 90% of group members correctly. Contrary to Sparks et al. (2022), not only did self-esteem fail to emerge as a significant predictor of incel status, it also loaded in the opposite direction. The two significant predictors were entered into their own regression equation where only perceived mate value emerged as a predictor of group membership. Finally, perceived mate value was entered as a lone predictor of group membership; this model was able to correctly classify 86% of participants.

**Table 6:** Variable correlations

Variable	1	2	3	4	5	6	7	8	9	10	11	12	13	14
1. Incel traits	–													
2. Fear of being single	0.691***	–												
3. Depression	0.708***	0.448***	–											
4. Anxiety	0.609***	0.439***	0.564***	–										
5. Self-esteem	−0.745***	−0.610***	−0.528***	−0.535***	–									
6. Secure attachment	−0.750***	−0.670***	−0.532***	−0.371***	0.606***	–								
7. Anxious attachment	0.301**	0.487***	0.189*	0.357***	−0.389***	−0.232**	–							
8. Avoidant attachment	0.369***	0.237**	0.267**	0.245**	−0.277***	−0.256**	−0.083	–						
9. Perceived mate value	−0.746***	−0.581***	−0.545***	−0.359***	0.616***	0.701***	−0.184*	−0.312***	–					
10. Sexual entitlement	0.180	0.162	0.044	0.059	−0.056	−0.134	0.115	0.125	−0.095	–				
11. External. of blame	−0.102	−0.236**	−0.186*	−0.096	0.278***	0.258**	−0.109	0.041	0.245**	0.276***	–			
12. Self-critical rumination	0.668***	0.591***	0.484***	0.599***	−0.685***	−0.482***	0.410***	0.208*	−0.424***	0.027	−0.125	–		
13. Perceived social support	−0.645***	−0.522***	−0.492***	−0.325***	0.501***	0.709***	−0.163	−0.329***	0.586***	−0.192*	0.225**	−0.400***	–	
14. Loneliness	0.708***	0.582***	0.617***	0.450***	−0.583***	−0.746***	0.347***	0.270***	−0.569***	0.111	−0.197*	0.538***	−0.684***	–

*** *p* < 0.001; ** *p* < 0.01; * *p* < 0.05

**Table 7:** Predictors of incel membership

	B	SE	aOR	95% CI for aOR	
Fear of being single	0.12	0.07	1.12	0.97	1.30
Depression	−0.02	0.10	0.98	0.80	1.19
Anxiety	−0.03	0.10	0.97	0.81	1.18
Self-esteem	0.55	0.30	1.73	0.96	3.12
Secure attachment	−0.17	0.11	0.84	0.68	1.04
Anxious attachment	0.16	0.10	1.17	0.97	1.4
Avoidant attachment	**0.17**	**0.08**	**1.18**	**1.01**	**1.39**
Mate value	−**0.37**	**0.10**	**0.69**	**0.57**	**0.85**
Self-critical rumination	0.03	0.07	1.03	0.91	1.17
Perceived social support	−0.06	0.04	0.94	0.87	1.02
Loneliness	−0.03	0.14	0.97	0.74	1.26
Model summary					
Cox & Snell *R*^*2*^	0.564				
Nagelkerke *R*^*2*^	0.774				
Avoidant attachment	0.08	0.05	1.08	0.98	1.19
Mate value	−**0.37**	**0.06**	**0.69**	**0.62**	**0.78**
Model summary					
Cox & Snell *R*^*2*^	0.466				
Nagelkerke *R*^*2*^	0.634				
Mate value	−**0.38**	**0.06**	**0.68**	**0.61**	**0.76**
Model summary					
Cox & Snell *R*^*2*^	0.458				
Nagelkerke *R*^*2*^	0.623				

Significant results are in bold

## Discussion

The present study sought to understand the role of loneliness, isolation, and social supports in the lives of incels. This was inspired by Maxwell et al. (2020) finding that social isolation is a key theme in incel discourse. The present study also sought to expand on a survey conducted in their analysis of r/Braincels paired with a survey conducted on the incels.me website where only one third of incels reported having at least one friend. If incels do experience solitude to a greater degree than non-incel men, this radically transforms and expands our understanding of incels and the context in which they experience romantic rejection (see Sparks et al., 2022 for a discussion on incels’ experiences of rejection). Results indicate that this is indeed the case, with incels reporting a higher degree of social and emotional loneliness and lower levels of social support from friends and family. Incels also demonstrated a pattern of using more problematic coping strategies, such as behavioural disengagement and self-blame relative to non-incel males, who had higher endorsement of healthier coping strategies, including seeking emotional support and positively reframing the situation. Similarly, incels reported much higher levels of self-critical rumination. These results support hypothesis 1, painting a relatively bleak picture of the means available to incels to express or cope with their frustrations, romantic or otherwise.

Not surprisingly, both loneliness and perceived social support correlated strongly with a number of mental and relational well-being items, such as depression, anxiety, self-esteem, fear of being single, secure attachment, and avoidant attachment, with anxious attachment only correlating with loneliness. Similar to Sparks et al. (2022), these well-being metrics were all domains where incels significantly differed from their non-incel counterparts, with incels demonstrating more anxious and depressive symptoms and anxious and avoidant attachment styles, while scoring lower in self-esteem and secure attachment similar to patterns observed by Costello et al. (2022a). While incels scored higher in measures of social dominance orientation, self-critical rumination, female sexual deceptiveness, and sexual entitlement (consistent with hypothesis 2), they actually scored lower on the externalization of blame measure (in contradiction with hypothesis 2). Regardless, these variables either did not or only sporadically correlated with the above variables (e.g., depressive symptoms, secure attachment) that showed moderate to large differences between the two groups. In its debut use with an actual incel sample, the Incel Traits scale did indeed correlate in the expected direction with the above variables where significant differences were found between incels and non-incel males (in support of hypothesis 3). A large, significant effect was found between scores on the Incel Traits scale with incels scoring 76% higher than non-incels.

When simultaneously entered into a binary logistic regression to predict group membership, only two variables emerged as significant, unique predictors of incel status: avoidant attachment and perceived mate value. This did not support hypothesis 4, which estimated that the lack of social supports and higher rates of loneliness would best differentiate incels from non-incels. When the two significant predictors were entered into their own binary logistic regression, only perceived mate value emerged as a significant predictor of incel group membership. Entered on its own, perceived mate value correctly classified 86% of participants, a high level of accuracy that did not meaningfully differ from the full model, which had a classification rate of 90%.

That neither social supports nor loneliness emerged as unique predictors of incel status is interesting, given that there were large differences found between incels and non-incels on these measures. It is even more curious given that they do not share a strong conceptual overlap with other variables that may have suppressed their predictive utility; however, there were strong correlations between both measures and other metrics that showed large effects such as secure attachment, depression, fear of being single, and perceived mate value. This statistical overlap may have contributed to social support and loneliness not emerging as unique predictors. It is important not to interpret these null findings as evidence that social supports and loneliness are not important elements of inceldom; rather, their contributions may be complex and involve other variables (e.g., lacking social supports may exacerbate depressive symptoms, feelings of loneliness may make their singlehood more salient).

### Implications

The novelty of the present study offers several suggestions to better understand the experiences of incels, an understudied group. Similar to Sparks et al. (2022), incels reported a pattern of increased rates—both in general and relative to their male comparisons—of mental health issues. These were moderately to strongly associated with incels’ lower levels of social support and higher feelings of loneliness. Although these were not uniquely predictive of being an incel, it highlights the potential importance of having social support, particularly in the face of romantic rejection (which incels experience to a large degree; Sparks et al., 2022), in potentially buffering against detrimental mental health outcomes. It also indicates that many of the qualitative works that have been done on incel forums that have either missed or minimized the loneliness theme that was identified by Maxwell et al. (2020), which may be key to the incel experience. Although it was expected that incels would report worse mental health, lower levels of social support, and greater perceptions of loneliness than their non-incel peers, what is particularly stunning is the magnitude of differences between the two groups given that data was collected in the midst of the COVID-19 pandemic—a period that has seen elevated rates of depression, anxiety, and isolation across general populations (Pfefferbaum & North, 2020; Pierce et al., 2020; Vigo et al., 2020; Wang et al., 2020). While the pandemic undoubtedly has a deleterious effect on the well-being of incels as well, it is possible that its effects actually *minimized* the differences between incels and non-incels. For instance, non-incels on averaged scored around the scale midpoint for loneliness, considerably higher than figures reported by individuals in France, Germany, and the Netherlands prior to the pandemic (De Jong Gierveld & Van Tillburg, 2010).

Despite the significant differences between incels and non-incels on virtually every metric, only two uniquely predicted incel group membership when all were entered simultaneously in a binary logistic regression: avoidant attachment and perceived mate value. This is somewhat similar to Sparks et al. (2022) where secure attachment and self-esteem emerged as the lone predictors of incel status. What is interesting in the present study is that self-esteem was close to emerging as a unique predictor (95% CIs: 0.96, 3.12), suggesting that it may be distinct from perceived mate value and that the latter is a more specific barometer for incels, as it is effectively a domain-specific measure of self-efficacy. That a form of attachment has again emerged as one of the few unique predictors of inceldom (low levels of secure attachment in Sparks et al. (2022), high levels of avoidant attachment in this study) suggests that this is a central feature that has been overlooked in academic and media analyses of incels. Given what *has* been discussed about incels, attachment researchers would likely not be surprised by the findings of the present study, as depictions of women as devious, conniving, and untrustworthy certainly reflect an insecure attachment style (Baele et al., 2021; Jones, 2020; O’Malley et al., 2020). This portrayal of women as shallow and unavailable is a somewhat ironic twist, given the reluctance of avoidantly attached persons to form close emotional bonds, which appears to be a central feature of incels.

The emergence of insecure attachment as a key issue among incels has been discussed primarily as a barrier to forming romantic or sexual relationships or as a potential contributor to incel views of women. However, it is also worth noting that their low levels of secure attachment in particular may also impact their platonic relationships with others and contribute to their feelings of loneliness and lack of social support. Indeed, strong relationships were found between secure attachment, loneliness, and social support. A recent longitudinal study by Loeb et al. (2021) has also found that attachment insecurity at age 14 is associated with later difficulties in receiving support from friends later in adolescence. Further, the relationship between insecure attachment and negative interactions with romantic partners was partially mediated by peer social support. This suggests that negative romantic interactions are the result of an interaction between insecure attachment and poor peer support, which may have implications for future incel research, particularly as involuntary celibacy researchers have long indicated that these issues are persistent and enduring over time (Donnelly et al., 2001).

One intriguing finding that emerged was the endorsement of the “venting” coping strategy, the lone healthy mechanism that incels utilized more frequently than their non-incel peers. Initially, this appears at odds to the other pattern of results, where incels report feeling alone and lacking social support, ostensible prerequisites to engage in venting. However, it is possible that this is referencing their use of incel and parallel forums as therapeutic spaces. In this vein, Helm et al. (2022) found that incels on Reddit often share their experiences and seek support through emotional expressions of frustration, loneliness, and hopelessness. That incels were recruited from incel-adjacent forums for this study strengthens this possibility. Multiple studies have indicated that online venting is not only common, but beneficial as well (Utz & Breuer, 2017; Vermeulen et al., 2018). There may even be social benefits; Wendorf and Yang (2015) found that online venting mediated the relationship between perceived stress and relationship maintenance, suggesting that online disclosure of personal problems may motivate individuals to invest more in their online friendships. An analysis of teenage Reddit use and mental health during the pandemic indicated that users were more likely to express more specific negative emotions and mental health issues in specific subreddits, suggesting that they may be more likely to share personal details in a community that may be more supportive or receptive to this information (Zhang et al., 2021). However, recent work by Himawan et al. (2021) on single men found that support received online was not associated with their life satisfaction or feelings of loneliness.

Despite these positive outcomes, it is important to note that Costello et al. (2022a) found that forum use actually predicted higher mean levels of anxiety. This has obvious relevance to the incel community, who saw their incel-specific subreddits, such as r/Incel, r/Braincel, and r/IncelWithoutHate shut down by Reddit over a period of three years. It also has implications for our interpretation of incel discourse on these forums. If incels lack real-world friends and social supports and are using online forums as avenues for the expression of negative emotions as a form of release or catharsis without regard for political (or even moral) correctness rather than rallying cries for an ideology, such a frame of reference must be taken into account when interpreting such discussions (see Neitzel & Welzer, 2012 and their use of reference frames when analyzing recorded conversation between National Socialist POWs in World War II).

### Limitations

The present study is not without limitations. While all incels self-identified as such, they were recruited through incel-adjacent subreddits. Attempts to recruit from some subreddits were forbidden by moderators, as was a request to advertise the study on incels.co. While the results of the present study were consistent with Sparks et al. (2022), which was able to recruit from incel-specific subreddits, it does limit the potential generalizability of the results. The study was also not advertised on 4chan, which some incels suggested may include more a more radical faction. How these individuals may differ from the incels in the present study is unknown, but worth future consideration. Similarly, the present study recruited incels through incel forums and some emerging research indicates that forum-using incels may differ ideologically from incels that do not engage with forums (Costello et al., 2022b). This, along with the smaller sample size of this hard-to-reach population means that readers should be careful not to generalize the results of this study to all incels. Another limitation of the present study was the measure used to capture social support; this is a well-validated measure in and of itself, but use of “friends” and “family” as constructs may have missed some of the support that incels may receive in their online communities (see their higher endorsement of venting as a coping mechanism). Given the anonymity of Reddit, it is possible that incels do receive support from their fellow incels, but do not consider them as “friends” in a traditional sense. Others have made this assertion with other Reddit communities, where “throwaway” accounts are created to discuss more stigmatized topics, affording the user support without exposing vulnerability (Ammari et al., 2019). However, another study exploring loneliness and life satisfaction among single men found that online support was not related to either outcome (Himawan et al., 2021).

### Future directions

Given that incels have repeatedly endorsed insecure forms of attachment, which have also been able to account for unique variance in predicting incel membership, it would be worthwhile investigating how these relate or even contribute to the depictions of women that incel forums have become infamous for. Such work could build on the foundation of Hart et al. (2012), who found that men’s benevolent and hostile sexism endorsement was associated with anxious attachment, while only the latter was associated with avoidant. Future research should also explore the role that social support and loneliness play in adopting the incel label and engaging in their forums. If indeed incels are using these spaces to seek support—which is the premise of the Reddit group r/IncelExit (for those seeking to leave the incel community)—because they lack supportive offline avenues for doing so, it would highlight a pressing need for mental health professionals to understand the unique circumstances of incels, who blend insecure attachment styles with internalizing behaviours and antisocial attitudes. Similarly, future work should explore the needs of incels in a therapeutic setting and what barriers exist to making this work efficacious. In addition to the higher rates of depression and anxiety found in the above two studies, a recent survey on incels.co found that despite prevalence rates in excess of 90% for both depression and anxiety, only half of incels reported seeking mental health support, with a meagre 6% reporting positive outcomes. It would thus be worthwhile using the present study’s results to inform a series of interviews and focus groups with incels that will identify their treatment needs, goals, and preferred approach. Also of clinical relevance is exploring whether incels have fixed or growth mindsets related to their mate value. To date, this is difficult to gauge as the Blackpill ideology—which many incels endorse—does not afford this possibility, yet discussions of *looksmaxxing* and *gymmaxxing* remain popular in incel forums.

### Conclusion

The present study was the first known study to quantitatively capture the social support and isolation elements of inceldom and their impact on incel livelihood. Results indicated that even during a global pandemic when mental health and isolation increased among the general public, incels still scored particularly worse on these than non-incel males. Mental and relational health variables that were both previously and presently associated with incels were related to self-reported rates of loneliness and social support. While these did not emerge as unique predictors of incel status, they do highlight the need for a broader understanding of incel experiences that captures not only their sexual exclusion, but their social isolation as well. These considerations should also inform clinical work with incels, who have reported low satisfaction with their previous attempts at therapy.

## Supplementary Information

Below is the link to the electronic supplementary material.

Supplementary file1 (DOCX 28 KB)

## Data Availability

The datasets generated and analysed during the current study are not publicly available, as participants did not provide consent for their data to be made available on a public repository. The datasets generated and analysed during the current study are available from the corresponding author on reasonable request.
