# Supplementary material for: One is the loneliest number: Involuntary celibacy (incel), mental health, and loneliness
Source: Curr Psychol. 2023 Feb 2:1–15. Online ahead of print. doi: 10.1007/s12144-023-04275-z (PMC9892684; doi:10.1007/s12144-023-04275-z)
Supplement: Supplementary file 1 — Supplementary file1 (DOCX 28 KB) [file 12144_2023_4275_MOESM1_ESM.docx]

# Supplemental Materials

At the request of reviewers, MANCOVAs were conducted with participant age, relationship status (single vs. dating/relationship), sexual orientation (heterosexual vs. sexual minority), ethnicity (European vs. others) education, political orientation, and dating app usage entered as covariates.

| **Table 2**  *Mental and Relational Health Responses* | | | | | |
| --- | --- | --- | --- | --- | --- |
|  | Incels | Non-incels |  |  |  |
|  | *M*(SD) | *M*(SD) | *F* | *d* | *n_p_*^2^ |
| Fear of being single | 25.20 (5.08) | 15.86 (6.77) | 43.662*** | 1.13 | .240 |
| Depression | 16.52 (4.16) | 12.62 (3.58) | 29.919*** | .94 | .178 |
| Anxiety | 18.74 (4.64) | 15.98 (4.30) | 19.456*** | .75 | .124 |
| Self-esteem | 2.50 (1.76) | 4.34 (1.58) | 36.492*** | 1.03 | .209 |
| Attachment |  |  |  |  |  |
| Secure | 8.13 (4.08) | 15.67 (4.16) | 61.772*** | 1.34 | .309 |
| Anxious | 16.02 (5.12) | 13.72 (4.49) | 6.857* | .45 | .047 |
| Avoidant | 11.44 (5.07) | 8.44 (4.33) | 15.886*** | .68 | .103 |
| *Note*. *** *p* < .001; ** *p* < .01; * *p* < .05 | | | | | |

| **Table 3**  *Responses to Rejection, Date-Related Attitudes, and Social Supports* | | | | | |
| --- | --- | --- | --- | --- | --- |
|  | Incels | Non-incels |  |  |  |
|  | *M*(SD) | *M*(SD) | *F* | *d* | *n_p_*^2^ |
| Perceived mate value | 10.73 (5.03) | 19.87 (3.73) | 100.708*** | 1.84 | .444 |
| Externalization of blame | 8.36 (5.99) | 9.81 (4.36) | .052 | .004 | .000 |
| Self-critical rumination | 33.27 (6.35) | 26.65 (7.56) | 24.789*** | .91 | .164 |
| Perceived social support | 25.66 (11.66) | 40.65 (10.67) | 40.365*** | 1.17 | .243 |
| Loneliness | 24.25 (3.44) | 18.77 (4.19) | 35.324*** | 1.09 | .219 |
| *Note*. *** *p* < .001; ** *p* < .01; * *p* < .05 | | | | | |

| **Table 4**  *Coping Strategy Endorsement* | | | | | |
| --- | --- | --- | --- | --- | --- |
|  | Incels | Non-incels |  |  |  |
|  | *M*(SD) | *M*(SD) | *F* | *d* | *n_p_*^2^ |
| Active coping | 5.04 (1.93) | 5.65 (1.83) | 1.688 | .23 | .013 |
| Planning | 5.66 (2.07) | 5.85 (1.90) | .145 | .07 | .001 |
| Positive reframing | 4.40 (2.16) | 5.44 (1.69) | 5.393* | .42 | .041 |
| Acceptance | 6.28 (1.73) | 6.31 (1.47) | .435 | .12 | .003 |
| Humour | 4.66 (2.33) | 5.33 (2.01) | 1.687 | .23 | .013 |
| Religion | 3.02 (1.61) | 3.52 (1.98) | 2.465 | .28 | .019 |
| Emotional support | 2.96 (1.35) | 4.46 (1.89) | 15.351*** | .71 | .109 |
| Instrumental support | 4.02 (2.07) | 4.45 (1.92) | .259 | .09 | .002 |
| Self-distraction | 6.43 (1.75) | 5.89 (1.73) | 1.815 | .24 | .014 |
| Denial | 3.15 (1.53) | 2.97 (1.72) | 3.468 | .34 | .027 |
| Venting | 5.02 (1.75) | 3.73 (1.54) | 19.104*** | .79 | .132 |
| Substance use | 4.19 (2.35) | 3.35 (1.90) | 3.384 | .33 | .026 |
| Behavioural disengagement | 5.09 (2.01) | 3.16 (1.64) | 26.035*** | .92 | .171 |
| Self-blame | 6.64 (1.55) | 5.25 (1.91) | 8.937*** | .54 | .066 |
| *Note*. *** *p* < .001; ** *p* < .01; * *p* < .05 | | | | | |

| **Table 5**  *Antisocial Attitudes* | | | | | |
| --- | --- | --- | --- | --- | --- |
|  | Incels | Non-incels |  |  |  |
|  | *M*(SD) | *M*(SD) | *F* | *d* | *n_p_*^2^ |
| Sexual entitlement | 11.61 (5.44) | 9.11 (3.72) | 11.475*** | .61 | .083 |
| Social dominance orientation | 15.80 (7.95) | 12.43 (6.68) | 4.814* | .40 | .037 |
| Belief in female sexual deceptiveness | 58.24 (16.35) | 42.52 (14.43) | 20.907*** | .83 | .141 |
| *Note*. *** *p* < .001; ** *p* < .01; * *p* < .05 | | | | | |

| **Table 6**  *Demographic Correlations* | | | | | | | | |
| --- | --- | --- | --- | --- | --- | --- | --- | --- |
| Variable | 1 | 2 | 3 | 4 | 5 | 6 | 7 | 8 |
| 1. Incel status | - |  |  |  |  |  |  |  |
| 2. Age | -.196** | - |  |  |  |  |  |  |
| 3. Relationship status | .395*** | -.007 | - |  |  |  |  |  |
| 4. Sexual orientation | .103 | .141 | -.039 | - |  |  |  |  |
| 5. Education | -.147* | .118 | -.004 | -.103 | - |  |  |  |
| 6. Ethnicity | -.109 | -.049 | -.101 | .104 | .012 | - |  |  |
| 7. Political orientation | .058 | -.121 | .006 | .077 | .068 | -.030 | - |  |
| 8. Dating app user | .268*** | -.129 | .283*** | -.145 | -.157* | -.119 | .047 | - |
| *Note*. Incel status (1 = incel, 2 = non-incel), relationship status (1 = single, 2 = dating/other), sexual orientation (1 = heterosexual, 2 = sexual minority), ethnicity (1 = non-European, 2 = European), dating app user (1 = yes, 2 = no)  *** *p* < .001; ** *p* < .01; * *p* < .05 | | | | | | | | |
